# Pharmacological manipulation of neurotransmitter activity induces disparate effects on cerebral blood flow and resting-state fluctuations

**DOI:** 10.1162/imag_a_00370

**Published:** 2024-11-20

**Authors:** Fanny Munsch, Manuel Taso, Daniel H. Wolf, Daniel Press, Stephanie Buss, John A. Detre, David C. Alsop

**Affiliations:** Division of MR Research, Department of Radiology, Beth Israel Deaconess Medical Center, Harvard Medical School, Boston, MA, United States; Institute of Bioimaging, University of Bordeaux, Bordeaux, France; Departments of Psychiatry and Neurology, University of Pennsylvania, Philadelphia, PA, United States; Department of Neurology, Beth Israel Deaconess Medical Center, Harvard Medical School, Boston, MA, United States

**Keywords:** arterial spin labeling, BOLD, brain connectivity, GABA, pharmacological MRI

## Abstract

Functional MRI methods can assess aspects of drug-induced brain response. Resting blood oxygenation level dependent (BOLD) fMRI and arterial spin labeling (ASL) perfusion MRI indirectly measure brain function through the coupling of activity to cerebral blood flow (CBF) and oxygenation but their relative sensitivity has not been directly compared. We assessed changes in resting measures of BOLD and ASL MRI in response to two neurotransmitter modulators: citalopram, a selective serotonin reuptake inhibitor, and alprazolam, a positive allosteric modulator of GABA type A receptor. Thirty healthy subjects were imaged in a placebo-controlled study, with N = 20 subjects receiving each treatment as part of an incomplete block design. Time-averaged CBF images from ASL and measures of resting-state fluctuations of BOLD and ASL images were assessed for significant effects. Following acute citalopram administration, analysis of the ASL data showed a reduction in time-averaged regional CBF in regions associated with high levels of 5-HT1A receptor density. In contrast, following alprazolam administration, BOLD amplitude of low-frequency fluctuations showed a highly significant and cortically widespread increase, consistent with the distribution of GABA-A receptors. Only a marginal decrease in ASL CBF was detected after alprazolam intake. BOLD and ASL are each sensitive to drugs targeting neurotransmitter systems, but appear to reflect different aspects of neural metabolism and the balance between excitatory and inhibitory activity. Accordingly, their combination may best capture the effects of neurotransmitter modulations, and thus be advantageous for pharmacological MRI studies.

## Abbreviations

**5-HT** 5-hydroxytryptamine

**ALFF** Amplitude of Low-Frequency Fluctuations

**ASL** Arterial Spin Labeling

**BOLD** Blood Oxygenation Level Dependent

**CBF** Cerebral Blood Flow

**CS** Compressed Sensing

**CSF** Cerebrospinal Fluid

**fALFF** fractional Amplitude of Low-Frequency Fluctuations

**fMRI** functional Magnetic Resonance Imaging

**FOV** Field Of View

**FSPGR** Fast Spoiled Gradient Echo

**GABA** Gamma Aminobutyric Acid

**IC** Intrinsic Connectivity

**LC** Local Correlation

**nuFFT** nonuniform Fast Fourier Transform

**pCASL** pseudo-Continuous Arterial Spin Labeling

**PET** Positron Emission Tomography

**phMRI** pharmacological Magnetic Resonance Imaging

**RARE** Rapid Imaging with Refocused Echoes

**ASL rsCBF fluctuations** Arterial Spin Labeling resting-state Cerebral Blood Flow fluctuations

**rsfMRI** resting-state functional Magnetic Resonance Imaging

**SS** Single-Shot

**SSRI** Selective Serotonin Reuptake Inhibitor

## 1 Introduction

Pharmacological modulation of neurotransmitter systems is a key therapeutic strategy that has been successful in alleviating symptoms of neurological and neuropsychiatric disease (Uysal, 2023). Combining pharmacological challenges with noninvasive functional imaging can potentially be used to determine regional target engagement of the drug, infer anticipated and unanticipated mechanisms of action, establish dose response, and provide markers of therapeutic response in clinical trials and clinical applications (Borsook et al., 2013;Carmichael et al., 2018;Jenkins, 2012;D. J. J. Wang et al., 2011). Such pharmacological MRI (phMRI) may also provide insights into the neurophysiological factors affecting functional MRI signals and their coupling to brain activity (Iadecola, 2017).

Two functional MRI methods have been widely employed for phMRI in humans: Arterial Spin Labeling (ASL) imaging (D. J. J. Wang et al., 2011), which directly quantifies regional cerebral blood flow (CBF), and Blood Oxygenation Level Dependent (BOLD) imaging (Wise & Tracey, 2006), which indirectly detects changes in regional CBF and metabolism. Both methods can detect transient functional changes induced by a task or stimulus. Although many phMRI studies have examined pharmacological manipulation of task activation, the interpretation of pharmacological effects in such studies is limited by the regions and networks involved in the applied tasks, the noise in measuring the drug effect as an interaction term between task and drug (Stewart et al., 2014), and the need to disentangle direct brain effects from indirect task performance effects (Canario et al., 2021).

BOLD and ASL can also detect task-independent transient changes in brain function, typically through fluctuations in regional signal changes and their temporal correlations with other brain regions, measures often referred to as resting-state fMRI (rsfMRI) (Canario et al., 2021). BOLD is much more widely used than ASL for these transient measures, but resting-state fluctuations in ASL CBF (ASL rsCBF fluctuations) can also be readily measured and quantified in physiological units with advanced acquisition methods (B. B. Biswal et al., 1997;Dai et al., 2016;Munsch et al., 2020). Administration of drugs can also have detectable effects on brain activity when averaged over many minutes and can be compared with other such images acquired many days or weeks apart. ASL time-averaged CBF is a more sensitive measure of such changes because BOLD sensitivity drops precipitously when comparing states widely separated in time (Aguirre et al., 2002;Stewart et al., 2014;J. Wang et al., 2003).

Different phMRI measures may reflect different characteristics of brain activity. Transient changes as seen in rsfMRI appear to be driven primarily by neurotransmitter release rather than metabolic activity (Iadecola, 2017;Zhu et al., 2022), but time-averaged CBF is increasingly coupled to metabolic activity on longer time scales (Iadecola, 2017;Lin et al., 2009;Mintun et al., 2002). The amplitude of fluctuations of local field potentials and EEG, and their correlations with other brain regions appear to be altered by the balance of regional excitatory and inhibitory activity (Ahmad et al., 2022;Gao et al., 2017), suggesting that fluctuation measures such as rsfMRI may also be sensitive to this balance (Larsen et al., 2022;Zhang et al., 2024;van Nifterick et al., 2024). Elevated excitatory/inhibitory ratio (E/I) has been hypothesized as a pathological mechanism in autism (Rubenstein & Merzenich, 2003) and Alzheimer’s disease (Fortel et al., 2022;Lauterborn et al., 2021), both disorders with reduced rsfMRI connectivity (Ibrahim et al., 2021;Lau et al., 2019). Others have suggested that higher rsfMRI functional connectivity in association cortices compared with sensory cortices may partially be explained by a higher E/I ratio (Fotiadis et al., 2023;Goulas et al., 2021). Differential modulation of inhibitory and excitatory activity also appears to change neurovascular coupling (Howarth et al., 2021;Lee et al., 2021;Uhlirova et al., 2016;Vo et al., 2023), potentially modulating the relative amplitude of CBF and BOLD responses (Buxton et al., 2014;Devi et al., 2022). This may, in part, reflect the existence of both excitatory and inhibitory pathways to control arteriolar dilation and contraction (Howarth et al., 2021;Kisler et al., 2017;Ruff et al., 2024;Uhlirova et al., 2016).

phMRI using drugs that can alter physiological factors such as excitation–inhibition ratio or neurovascular coupling, in addition to changing time-averaged metabolism, can be used to provide insights into the relative sensitivity of functional imaging methods to these different factors. In this study, we compared task-independent ASL and BOLD signal changes in response to pharmacological modulation with drugs targeting two different neurotransmitter systems. We hypothesized that the effect size and spatial distribution of measured brain response to pharmacological challenge would be different for each drug and between BOLD rsfMRI and ASL time-averaged CBF. We chose two widely studied and prescribed neuroactive drugs: citalopram, a serotonin reuptake inhibitor, and alprazolam, a GABA-positive allosteric modulator, because they have previously been used in pharmacological MRI studies and target different neurotransmitter systems. Alprazolam administration has previously been proposed as a model for excitation–inhibition ratio modulation because of its strong and widespread inhibitory effects (Larsen et al., 2022;Zhang et al., 2024). We also hypothesized that the spatial distribution of measured brain responses should overlap with the spatial distribution of these neurotransmitters. We compared the changes in BOLD rsfMRI and ASL time-averaged CBF after acute oral administration of these drugs in healthy volunteers. We also assessed ASL rsCBF fluctuations metrics derived from the ASL time series data. Comparison of responses between these fMRI methods can inform the design and interpretation of future pharmacological studies and provides insights into which physiological factors contribute most to the functional sensitivity.

## 2 Methods

### 2.1 Study design

Studies were performed in N = 30 volunteers (17 females; ages ranging from 19 to 42 years) recruited as part of a double-blinded study according to a protocol compliant with state and federal regulations governing the conduct of human subject research (45 Code of Federal Regulations (CFR) Part 46 and 21 CFR Parts 50 and 56), adhering to the ethical principles set forth in the Belmont Report and approved by the Beth Israel Deaconess Medical Center Committee on Clinical Investigations (protocol number 2014P000381). Written informed consent was obtained from all participants. All participants were free from major physical or mental health conditions as confirmed by a physical examination and blood testing prior to the scan days. The participation involved two scanning sessions at least 2 weeks apart, and the participants were randomized to receive two out of three potential treatments: placebo, citalopram (20 mg), and alprazolam (0.5 mg), in an incomplete block design with treatment and order of treatment fully permuted in blocks of six and the order of scans (ASL vs. BOLD) reversed every other block. Every treatment was thus administered to N = 20 participants. Drug treatments were randomly assigned a number by the research pharmacy so all staff and participants were blinded to treatment until after analysis. The protocol included a baseline MRI examination in the morning followed by the administration of the drug. A second identical MRI examination was performed 4 h after the baseline scan and 2 h after the administration of the drug consistent with the drug pharmacokinetics. Two participants were excluded due to poor labeling efficiency of their ASL acquisitions. Thus, N = 28 participants completed all study procedures and were included in final analyses.

### 2.2 MRI acquisition

The participants were scanned on a 3T scanner (Discovery MR750, GE Healthcare, Waukesha, WI), using body coil RF transmission and a 32-channel head coil for reception (Nova Medical, Wilmington, MA). Cardiac and respiratory fluctuations were digitally recorded from pulse oximetry and a respiratory bellow during the scan. These signals were converted to cardiac and respiratory rates at the time of scan by determining the peak after Fourier transformation. Their potential influence on BOLD signal after different drug intake was assessed with a linear mixed-effect model defining the respiratory or cardiac rate as the outcome variable, the conditions (e.g., alprazolam_post_— placebo_post_) as a fixed effect and subjects as a crossed random effect. Acquisitions included a 1 mm isotropic 3D T1-w FSPGR sequence and an ASL sequence using a golden-angle Stack-of-Spirals RARE trajectory (Munsch et al., 2020), with 13 interleaves rotated by an approximate golden angle (10 π/13) of a 1,536 points spiral waveform (TR/TE = 6,574/12.9 ms, receiver bandwidth = 125 kHz, echo spacing 12 ms, individual spiral waveforms of 6.1 ms). Thirty-two centrically ordered, 4-mm thick slices were acquired within an acquisition time of 9 min. Slice encodes were also rotated by the approximation of the golden angle. Label and control images were interleaved followed by rotations of the spiral encode patterns after each pair. In total, 39 excitations, meaning 39 single-shot ASL volumes, were acquired corresponding to 3 averages of 13 rotations. A 13 interleave M_0_reference image with presaturation at 2 s before imaging was also acquired at the end of the sequence. The ASL preparation relied on a background-suppressed unbalanced pseudo-continuous ASL (pCASL) scheme (1.8 s labeling, 1.8 s postlabeling delay) with interleaved labeling and background suppression pulses. Labeling was performed at the level of the C2–C3 intervertebral disk (Zhao et al., 2017). Errors in labeling efficiency occurred in two participants for the ASL acquisition, causing the exclusion of the ASL data from these two participants (N = 28).

We also acquired BOLD rsfMRI using a multiband 2D Gradient-echo Echo-Planar-Imaging sequence with the following parameters: 500 volumes of 66 axial slices; TR, 900 ms; TE, 25 ms; matrix resolution, 108 x 108; FOV, 216 x 216 mm^2^; multiband acceleration, 6 and slice thickness, 2 mm. Scans were quality assessed with visual and mean signal methods. Errors in acquisition appeared for two subjects for whom only 499 and 488 volumes were retained for the analysis (multiband artifacts for both data and missing volumes for the latter one).

### 2.3 Image reconstruction and analyses

#### 2.3.1 ASL image reconstruction

Raw ASL data were first preprocessed to reduce slice direction blurring (Zhao et al., 2017) and Gibbs ringing. Data were corrected for signal decay between echoes by multiplying each echo with a correction factor derived from simulations of the reduced flip angle RARE sequence and assuming T_2_= 100 ms and T_1_= 1,400 ms. A spherical Fermi type filter (Zhao et al., 2017) was also applied to control Gibbs ringing and reduce noise contribution from the corners of k-space. Furthermore, as the employed Fermi filter was not sufficient to remove all ringing artifacts present in the reference images, a Hann window was also applied to the reference raw data.

Reconstruction of ASL images followed two separate paths. For time-averaged CBF image reconstruction, all the data were combined prior to reconstruction to create fully sampled ASL perfusion-weighted and proton density-weighted reference images that were then reconstructed using the BART toolbox (Uecker, et al., 2015) by performing a 3D nonuniform Fast Fourier Transform (nuFFT) with L_2_regularization (λ = 0.01) followed by phased coil combination (Bydder et al., 2002) of the 32 individual channels. Separately, the 39 individual golden-angle rotations were reconstructed as undersampled single-shot (SS) perfusion-weighted volumes using an L_1_-wavelet regularized Compressed Sensing (CS) reconstruction of the complex subtracted data as previously described (Munsch et al., 2020).

#### 2.3.2 Anatomical and ASL processing and registration

T1-weighted images were segmented using the default SPM12 (Statistical Parametric Mapping, Welcome Trust Center for Neuroimaging, London, UK) segmentation options to produce gray matter, white matter, and CSF masks. These images were calculated in the original space and, in addition, the spatial transformations to register to MNI152 space were determined.

To correct for any motion between the perfusion weighted and reference images, registration of the reference image to the time-averaged perfusion weighted image was performed. Since registration of the raw reference image sometimes caused errors due to the different image contrast, a perfusion-like image was created from the reference image by first segmenting the images within SPM and then adding the gray matter probability map to half the CSF and white matter probability maps. Registration parameters were determined using the perfusion-like image as the target. This coregistered reference image was used for both mean and SS time series CBF quantification. Finally, quantification was performed by calculating CBF maps from both the time-averaged perfusion weighted and individual SS perfusion-weighted images, in voxels whose intensity in the reference image was higher than 65% of the mean bias corrected WM signal (calculated from a bias corrected reference image which was dilated with 3D 3 voxel-wide cubic structuring element), using a two-compartment model (Parkes, 2005). This threshold has been chosen to avoid some sharp cutoff at the edge of the brain.

Globally normalized versions of the SS time series CBF and time-averaged CBF images were created by scaling the CBF images to achieve a fixed 50 mL/100 g/min whole brain average. This global normalization removes differences in whole brain average across time and subjects. Both globally normalized and unnormalized time-averaged CBF images were assessed for drug effects. Only globally normalized SS time series CBF images were used to determine resting-state fluctuations of CBF.

Spatial transformation to a standard anatomical space was also performed. The time-averaged CBF images were coregistered to the gray matter probability map calculated from the T1-weighted anatomical images and then transformed to MNI152 space using the parameters determined from the anatomical images.

All these steps were performed using SPM12 and custom MATLAB (R2020b, MathWorks, Natick, MA) scripts.

#### 2.3.3 BOLD fMRI processing and registration

BOLD rsfMRI preprocessing was performed with the CONN toolbox (Whitfield-Gabrieli & Nieto-Castanon, 2012) using the default preprocessing pipeline (functional realignment and unwarping, structural segmentation and normalization, functional normalization, outlier detection, and smoothing), after removing the first five volumes which were affected by transient effects. Then, a denoising step was performed by applying a linear regression and band-pass filtering ([0.008–0.09 Hz]) to remove unwanted motion, physiological, and other artifactual effects from the BOLD signal before computing connectivity measures. Two different sources of possible confounders were defined: (1) BOLD signal from the white matter and CSF masks and (2) any previously defined within-subject covariate (realignment and scrubbing parameters). The realignment covariate is a set of six movement parameters representing movement in the three translational and rotational directions. Scrubbing involves removing a variable number of noise components (e.g., outlier scans) which are used as potential confounding effects to remove their potential influence on the BOLD signal. The motion parameters from the realignment were further processed to obtain a quantitative estimate for motion during each time series. After detrending of each of the three translational and three angular motion parameters, the temporal standard deviation was calculated, the angular standard deviations in radians were multiplied by an effective radius of 80 mm, and then the root mean square of the six motion parameters was calculated as an overall motion measure. The difference in motion between drug states and placebo was assessed with a linear mixed-effect model defining the overall motion measure as the outcome variable, the conditions as a fixed effect and subjects as a crossed random effect.

#### 2.3.4 Calculation of individual spontaneous fluctuation maps for both BOLD and ASL time series

To assess altered spontaneous fluctuations changes after pharmacological manipulation, we used the CONN toolbox (Whitfield-Gabrieli & Nieto-Castanon, 2012) for both ASL rsCBF fluctuations and BOLD rsfMRI analyses. The default parameters were applied for both techniques, except for some applicable to only BOLD signal: CSF, WM, and effect of rest as confounds in denoising. The first level analysis consisted of computing voxel-level measures of global and local connectivity patterns. BOLD percent signal change was used as the analysis unit.

To study local fluctuation changes, we computed amplitude of low-frequency fluctuations (ALFF) maps (Yu-Feng et al., 2007) and local correlation (LC) (Deshpande et al., 2009), while we computed intrinsic connectivity (IC) (Martuzzi et al., 2011) to assess more global connectivity patterns. These measures are widely used and support whole brain analyses similar to time-averaged ASL CBF without*a priori*hypotheses about the spatial or temporal characteristic of the response. ALFF was measured within the 0.01–0.08 Hz range, though the lower temporal sampling of the SS ASL images (13 s) limited the maximum frequency to 0.038 Hz. Low-frequency fluctuations are assumed to reflect spontaneous neural activity of the brain (B. Biswal et al., 1995). LC is a measure of local coherence at each voxel, which characterizes the average correlation between each voxel and its neighbors, here defined within a 25 mm local kernel window. IC is a measure of network centrality at each voxel, which characterizes the strength of the connectivity between each voxel and the rest of the brain.

#### 2.3.5 Image-based analyses

We performed a voxel-wise general linear model within CONN and SPM12 to compare drug effect on the brain between the drug group (citalopram or alprazolam), N = 20 participants, and the placebo group, N = 20 participants, for time-averaged CBF and each of the three fluctuation measures for both ASL rsCBF fluctuations and BOLD rsfMRI. As previously shown (Munsch et al., 2021), while baseline scans before the administration of drug are not required for designs of resting ASL analyses (time-averaged CBF), incorporating them may increase power to detect an effect by controlling for physiological and potentially experimental effects across days. We used the term “controlled by morning” to refer to the use of the morning scan. Thus, the contrast time-averaged CBF_post–pre__drug_< CBF_post–pre__placebo_was assessed too. Two-sided cluster-level T-contrasts were obtained using nonparametric statistics (1,000 randomizations of the signs of the residuals). Type I error was controlled using voxel-level and cluster-level thresholds (respectively, p < 0.01 uncorrected and p < 0.05 cluster-mass false discovery rate corrected).

#### 2.3.6 Regional analyses

To increase statistical power and provide specificity to neurotransmitter hypotheses, we also performed analyses using values derived by weighted summation of signals over neurotransmitter-related density maps. We used publicly available PET-based human atlases of the serotonergic system (Beliveau et al., 2017), the target of citalopram, (the 5-HTT serotonergic transporter and 5-HT1A, 5-HT1B, 5-HT2A, and 5-HT4 serotonin receptors), and the distribution of the GABA-A receptor (Nørgaard et al., 2021), the target of alprazolam (Supplementary Fig. 1). Using a dedicated MATLAB program, we computed a total weighted factor, for every scan of every subject, which was calculated by summing voxel wise the voxel value (time-averaged CBF, ALFF, IC, or LC) multiplied with the corresponding neurotransmitter density value. Next, within R (version 4.2.0), we fit a linear mixed-effect model defining the weighted factor calculated for each receptor/transporter as the outcome variable, the conditions as a fixed effect and subjects as a crossed random effect. Significant results were defined with p < 0.008 (0.05 Bonferroni corrected for the six density maps).

## 3 Results

All subjects tolerated the medications and scanning without incident and were cleared to leave shortly after the last scan. Motion during the scans was not significantly different for the medicated scans relative to placebo (Supplementary Table 1). Cardiac and respiratory rates were not significantly different for the three treatments (Supplementary Table 1).

### 3.1 Effects on brain blood flow and resting-state fluctuations after citalopram administration

#### 3.1.1 Citalopram significantly reduces ASL time-averaged CBF in a region surrounding the amygdala and within 5-HT1A, 5-HT4, and 5-HTT density-weighted regional analysis

**Fig. 1.**
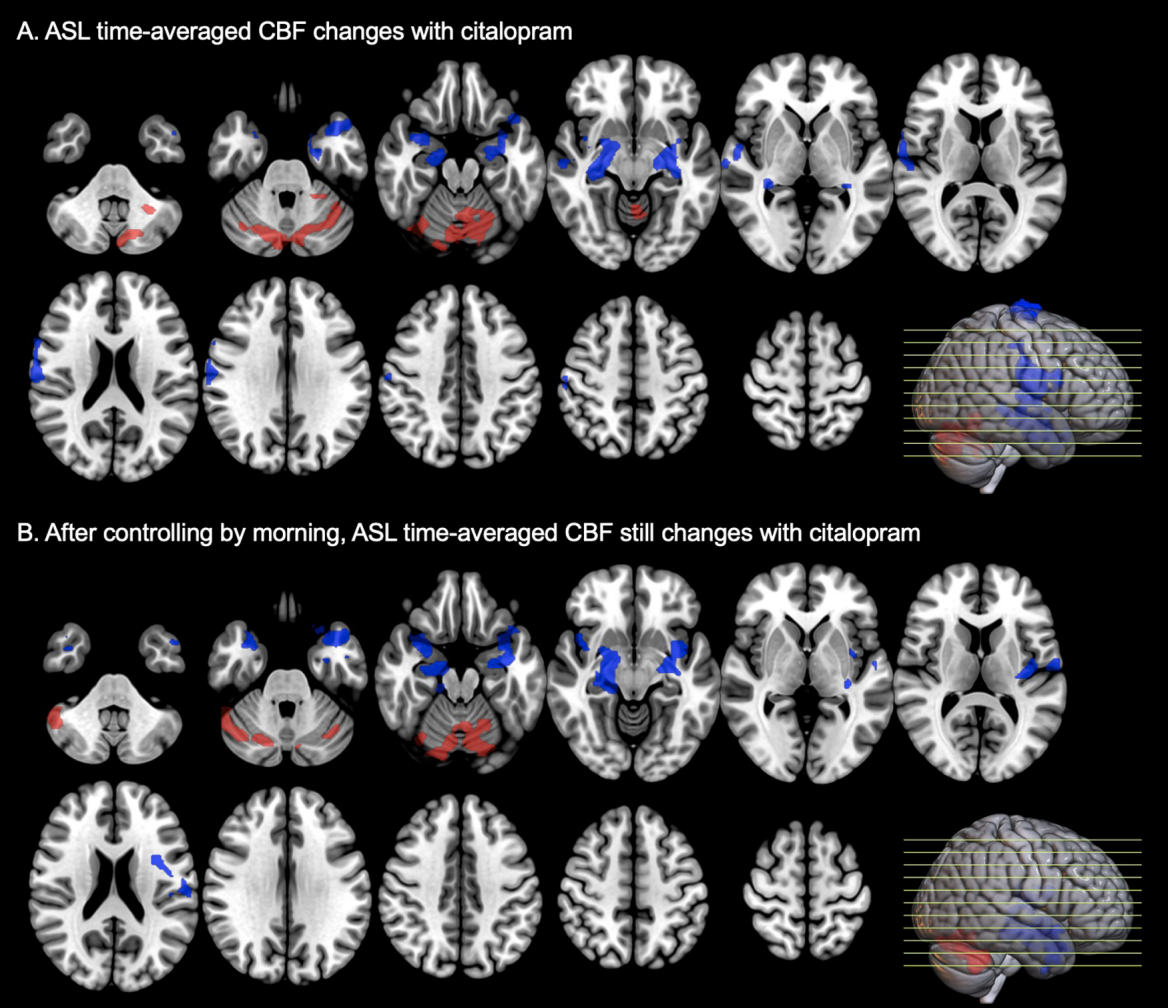
Significant globally normalized ASL time-averaged CBF changes in the citalopram group (N = 20) compared with the placebo group, even after controlling by morning—Note: red means signal increase, blue means signal decrease, both ROIs are binarized after voxel-level and cluster-level thresholding (respectively, p < 0.01 uncorrected and p < 0.05 cluster-mass false discovery rate corrected).

Significant mean globally normalized ASL time-averaged CBF_post_changes between the drug and the placebo were detected with voxel-based analysis after administration of citalopram (Fig. 1;Supplementary Table 2).

A significant mean globally normalized ASL time-averaged CBF decrease in both amygdala, hippocampi, insula, some parts of the temporal lobes, and the supplementary motor area (SMA), and mean globally normalized ASL time-averaged CBF increase in the cerebellum occurred after administering citalopram (Fig. 1A;Supplementary Table 2). As previously reported (Munsch et al., 2021), controlling by the first examination (here, morning) increased the significance of results when assessing mean globally normalized ASL CBF time-averaged changes between two time points with image-based analyses (Fig. 1B;Supplementary Table 3).

Results from image-based analyses were confirmed by the neurotransmitter targeted regional analyses. The highest significance was seen for 5-HT1A receptor weighting, followed closely by 5-HT4 and then 5-HTT. However, controlling by the morning examination reduced the significance of ROI-based analyses, but general patterns were similar (Table 1).

**Table 1.** Globally normalized ASL time-averaged CBF changes with citalopram intake in neurotransmitters regions.

Neurotransmitter receptors/transporters	Conditions	z value	p-value
5-HTT	Citalopram *_post_* > placebo *_post_*	-2.95	**0.003**
	Citalopram *_post–pre_* > placebo *_post–pre_*	-1.76	0.078
5-HT1A	Citalopram *_post_* > placebo *_post_*	-3.96	**7e-05**
	Citalopram *_post–pre_* > placebo *_post–pre_*	-2.65	**0.008**
5-HT1B	Citalopram *_post_* > placebo *_post_*	-2.10	0.036
	Citalopram *_post–pre_* > placebo *_post–pre_*	-0.50	0.615
5-HT2A	Citalopram *_post_* > placebo *_post_*	-2.24	0.025
	Citalopram *_post–pre_* > placebo *_post–pre_*	-0.69	0.490
5-HT4	Citalopram *_post_* > placebo *_post_*	-3.48	**5e-04**
	Citalopram *_post–pre_* > placebo *_post–pre_*	-1.97	0.049
GABA-A	Citalopram *_post_* > placebo *_post_*	0.38	0.706
	Citalopram *_post–pre_* > placebo *_post–pre_*	1.54	0.124

Linear mixed-effect model assessing the relationship between mean globally normalized ASL CBF and neurotransmitters receptors/transporters density profiles after citalopram intake. p-values highlighted in bold are significant after Bonferroni correction for multiple comparisons.

When the analysis was performed without global normalization of ASL time-averaged CBF, no significant effects of citalopram were detected, likely due to variability in global CBF, reducing the sensitivity to relative regional differences (Supplementary Table 4).

#### 3.1.2 No significant effects of citalopram on resting-state fluctuations, measured with ALFF, IC, and LC, were detected with BOLD rsfMRI or ASL rsCBF fluctuations

In contrast to time-averaged CBF findings described above, all image-based and region-based analyses of ASL rsCBF fluctuations and BOLD rsfMRI failed to show significant drug effects (Supplementary Tables 5–10).

### 3.2 Alprazolam increases resting-state BOLD rsfMRI ALFF and, to a lesser extent, network correlations

#### 3.2.1 A highly significant effect of alprazolam on BOLD rsfMRI ALFF was detected but no change in ASL rsCBF fluctuations ALFF was observed

**Fig. 2.**
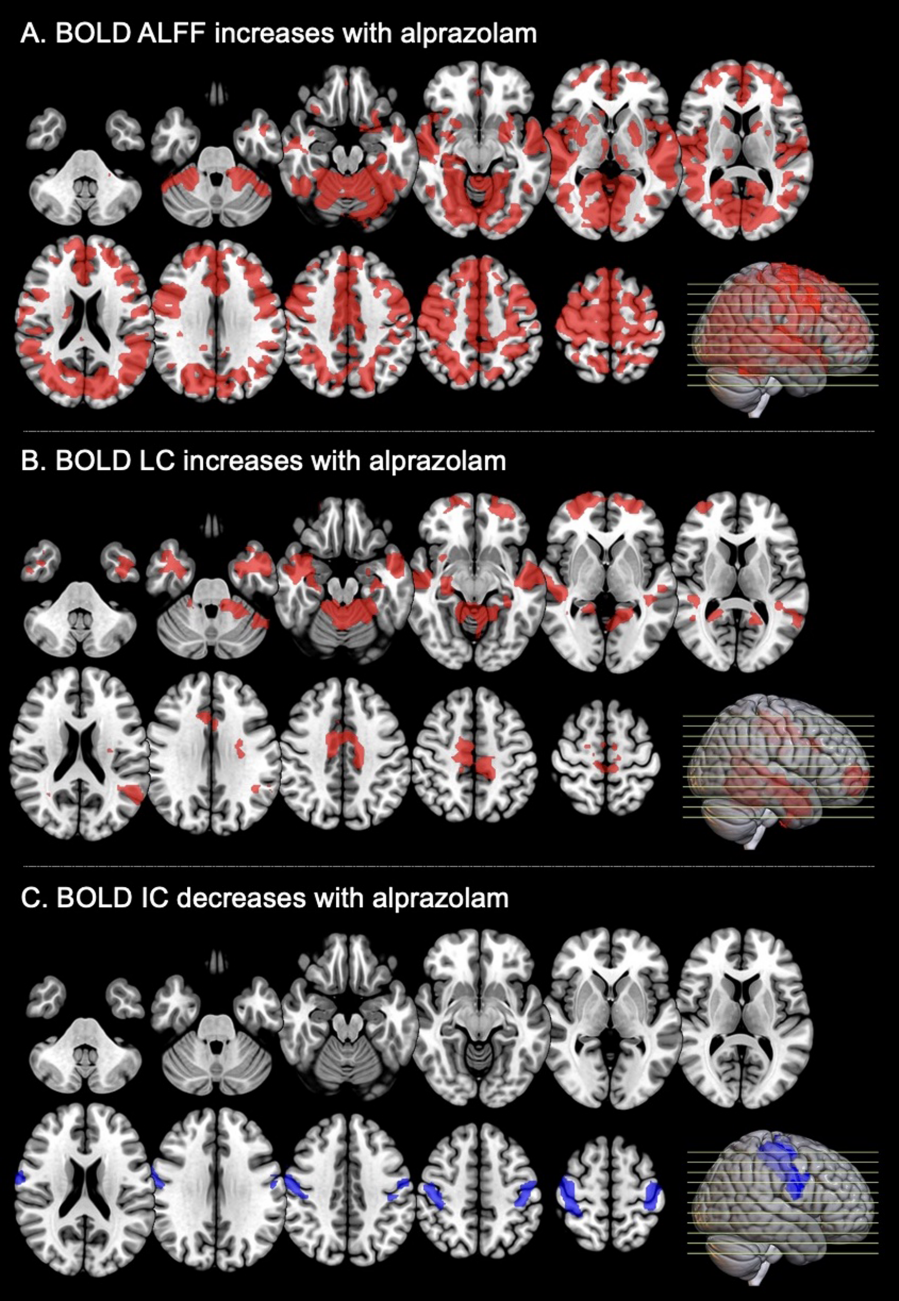
All BOLD rsfMRI metrics changes in the alprazolam group (N = 20) compared with the placebo group in the afternoon examination. Note: ALFF = amplitude of low-frequency fluctuations; LC = local correlation; IC = intrinsic connectivity/red means signal increase, blue means signal decrease, both ROIs are binarized after voxel-level and cluster-level thresholding (respectively, p < 0.01 uncorrected and p < 0.05 cluster-mass false discovery rate corrected).

An increase of the amplitude of BOLD signal fluctuations, measured with ALFF, was observed across almost the entire neocortex after alprazolam intake with both image-based and regional analyses (Fig. 2A;Table 2).

**Table 2.** BOLD rsfMRI ALFF changes with alprazolam intake in neurotransmitters regions.

Neurotransmitter receptors/transporters	Conditions	z value	p-value
5-HTT	Alprazolam *_post_* > placebo *_post_*	3.66	**2e-04**
	Alprazolam *_post–pre _* > placebo *_post–pre_*	3.08	**0.002**
5-HT1A	Alprazolam *_post_* > placebo *_post_*	3.74	**2e-04**
	Alprazolam *_post–pre_* > placebo *_post–pre_*	3.20	**0.002**
5-HT1B	Alprazolam *_post_* > placebo *_post_*	3.83	**1e-04**
	Alprazolam *_post–pre_* > placebo *_post–pre_*	3.24	**0.001**
5-HT2A	Alprazolam *_post_* > placebo *_post_*	3.81	**1e-04**
	Alprazolam *_post–pre_* > placebo *_post–pre_*	3.25	**0.001**
5-HT4	Alprazolam *_post_* > placebo *_post_*	3.80	**1e-04**
	Alprazolam *_post–pre_* > placebo *_post–pre_*	3.25	**0.001**
GABA-A	Alprazolam *_post_* > placebo *_post_*	3.81	**1e-04**
	Alprazolam *_post–pre_* > placebo *_post–pre_*	3.25	**0.001**

Linear mixed-effect model assessing the relationship between BOLD rsfMRI ALFF maps and neurotransmitters receptors/transporters density profiles after alprazolam intake. p-values highlighted in bold are significant after Bonferroni correction for multiple comparisons.

Fully detailed brain regions where global signal fluctuations increased significantly are given inSupplementary Table 11. Since the ALFF increase was so significant and widespread, it was, unsurprisingly, significant within all serotonergic receptors/transporter and GABA-A receptor-weighted regions.

In contrast to the widespread increase in BOLD rsfMRI ALFF, ASL rsCBF fluctuations ALFF showed no significant change on either image-based or neurotransmitter-weighted regional analyses (Supplementary Table 12).

#### 3.2.2 Increases with alprazolam in BOLD rsfMRI and ASL rsCBF fluctuations connectivity measures were detected

Though not as significant as the BOLD rsfMRI ALFF change, increased BOLD rsfMRI LC was observed in some areas on both the image-based analysis (Fig. 2B) and the neurotransmitter regional analyses (Table 3). Elevated LC was present in the image-based analysis within some temporal and frontal regions (significant regions fully described inSupplementary Table 13) and reached significance in 5-HTT, 5-HT1A, and 5-HT4 density-weighted regional analysis (Table 3).

**Table 3.** BOLD rsfMRI LC changes with alprazolam intake in neurotransmitters regions.

Neurotransmitter receptors/transporters	Conditions	z value	p-value
5-HTT	Alprazolam *_post_* > placebo *_post_*	3.06	**0.002**
	Alprazolam *_post–pre_* > placebo *_post–pre_*	2.59	*0.010*
5-HT1A	Alprazolam *_post_* > placebo *_post_*	3.00	**0.003**
	Alprazolam *_post–pre_* > placebo *_post–pre_*	2.48	*0.013*
5-HT1B	Alprazolam *_post_* > placebo *_post_*	2.58	*0.010*
	Alprazolam *_post–pre_* > placebo *_post–pre_*	2.12	0.034
5-HT2A	Alprazolam *_post_* > placebo *_post_*	2.55	*0.011*
	Alprazolam *_post–pre_* > placebo *_post–pre_*	2.13	0.033
5-HT4	Alprazolam *_post_* > placebo *_post_*	3.00	**0.003**
	Alprazolam *_post–pre_* > placebo *_post–pre_*	2.46	*0.014*
GABA-A	Alprazolam *_post_* > placebo *_post_*	2.48	*0.013*
	Alprazolam *_post–pre_* > placebo *_post–pre_*	2.23	0.026

Linear mixed-effect model assessing the relationship between BOLD rsfMRI LC maps and neurotransmitters receptors/transporters density profiles after alprazolam intake. p-values highlighted in bold are significant after Bonferroni correction for multiple comparisons.

No significant increased effects were found for BOLD rsfMRI IC analyses (Supplementary Table 14) and a decrease of IC in primary motor areas was observed (Fig. 2C), as detailed inSupplementary Table 15.

**Fig. 3.**
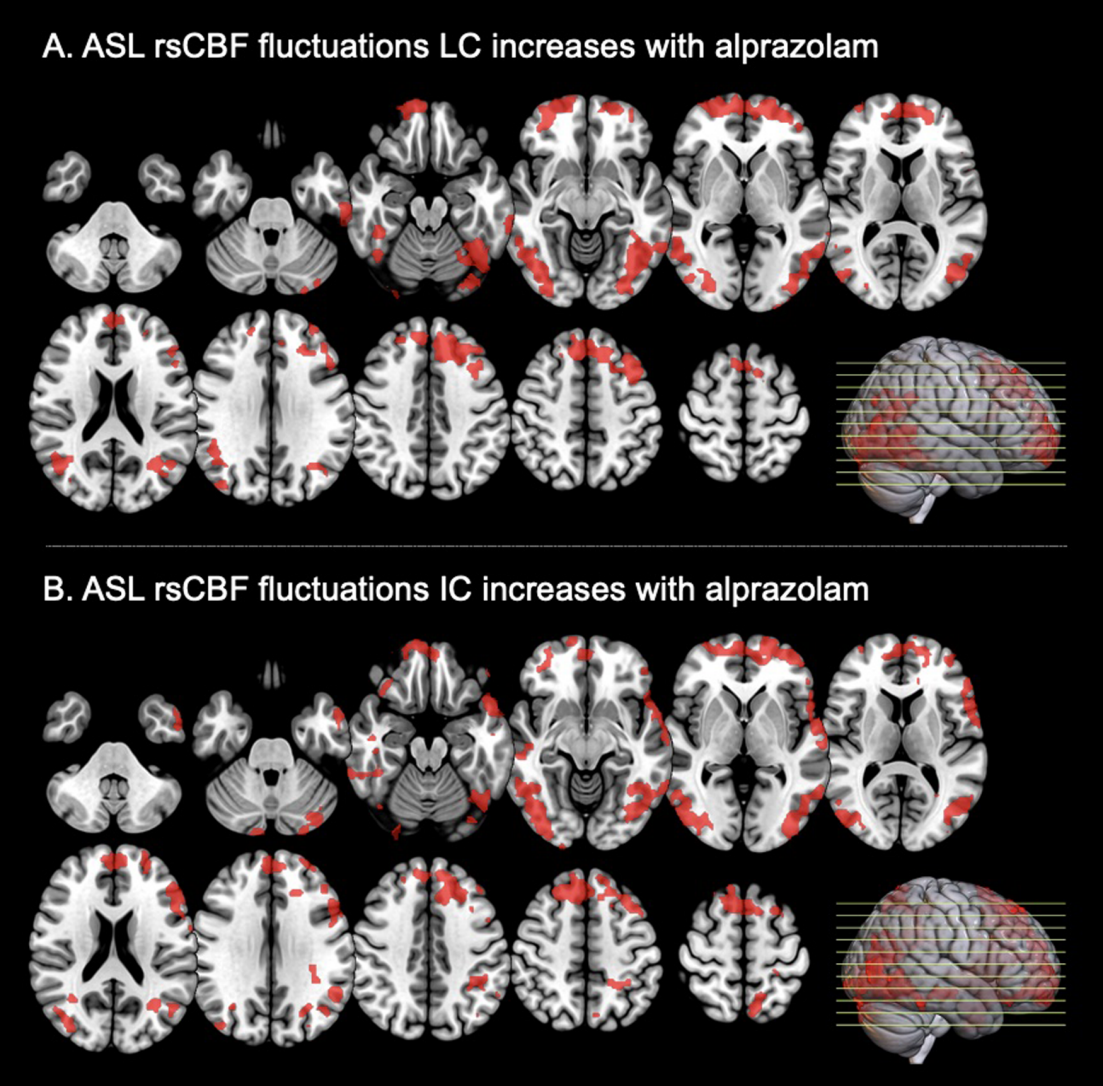
ASL rsCBF fluctuations LC and IC changes in the alprazolam group (N = 20) compared with the placebo group (N = 20) in the afternoon examination. Note: LC = local correlation; IC = intrinsic connectivity/red means signal increase, blue means signal decrease, both ROIs are binarized after voxel-level and cluster-level thresholding (respectively, p < 0.01 uncorrected and p < 0.05 cluster-mass false discovery rate corrected).

In the ASL rsCBF fluctuations analyses, increased LC and IC were observed, as shown inFigure 3. A significant increase of the correlation measures after alprazolam intake can be seen in frontal, orbitofrontal, temporal, and parietal regions with ASL rsCBF fluctuations (Fig. 3).

Significant brain regions are fully described inSupplementary Table 16for LC andSupplementary Table 17for IC. However, the neurotransmitter-weighted ROIs analyses did not reach significance for LC (Supplementary Table 18) and IC (Supplementary Table 19).

#### 3.2.3 Marginal decrease of mean globally normalized time-averaged CBF induced by alprazolam

While image-based analyses of time-averaged CBF either with or without global normalization did not reach significance after alprazolam intake, neurotransmitter-weighted ROI analyses of globally normalized time-averaged CBF data showed a marginal decrease of time-averaged CBF (Table 4) with the greatest effect in the 5-HT2A ROI, which emphasizes mostly cortex but deemphasizes subcortical and cerebellar regions. The nonsignificant results for time-averaged CBF data (without global signal normalization) are given inSupplementary Table 20.

**Table 4.** Globally normalized time-averaged CBF changes with alprazolam intake in neurotransmitters regions.

Neurotransmitter receptors/transporters	Conditions	z value	p-value
5-HTT	Alprazolam *_post_* > placebo *_post_*	-0.26	0.795
	Alprazolam *_post–pre_* > placebo *_post–pre_*	-0.41	0.680
5-HT1A	Alprazolam *_post_* > placebo *_post_*	-2.56	0.010
	Alprazolam *_post–pre_* > placebo *_post–pre_*	-2.72	**0.007**
5-HT1B	Alprazolam *_post_* > placebo *_post_*	-2.63	0.009
	Alprazolam *_post–pre_* > placebo *_post–pre_*	-2.45	0.015
5-HT2A	Alprazolam *_post_* > placebo *_post_*	-2.88	**0.004**
	Alprazolam *_post–pre_* > placebo *_post–pre_*	-2.72	**0.007**
5-HT4	Alprazolam *_post_* > placebo *_post_*	-1.63	0.104
	Alprazolam *_post–pre_* > placebo *_post–pre_*	-2.21	0.027
GABA-A	Alprazolam *_post_* > placebo *_post_*	-1.79	*0.073*
	Alprazolam *_post–pre_* > placebo *_post–pre_*	-0.27	0.785

Linear mixed-effect model assessing the relationship between globally normalized time-averaged CBF and neurotransmitters receptors/transporters density profiles after alprazolam intake. p-values highlighted in bold are significant after Bonferroni correction for multiple comparisons.

## 4 Discussion

Our findings demonstrate that phMRI using BOLD rsfMRI and ASL provides strikingly different findings across modalities after pharmacological modulation of two different neurotransmitter systems. With administration of the selective serotonin inhibitor citalopram, the analysis of the ASL data showed a highly significant reduction in time-averaged regional CBF in regions associated with serotonin receptor densities for 5-HT1A and 5-HT4, while BOLD rsfMRI changes did not even approach significance. In contrast, administration of the GABA-A receptor-positive allosteric modulator alprazolam induced a highly significant increase in BOLD fluctuation amplitude, as measured by ALFF, across the cortex. More modest but significant increases in BOLD local correlations were also observed. Only a marginal decrease in ASL CBF was observed after alprazolam and ASL rsCBF fluctuations were not increased in amplitude, though increases in local and intrinsic connectivity were detected. These differences likely reflect both mechanistic differences between the two drugs and the differential sensitivities of BOLD rsfMRI and ASL to their underlying mechanisms.

Neurophysiological differences between the two drugs and the neurotransmitter systems they target can explain some of the differences in relative responses. Serotonergic signaling originates in the raphe nuclei, distant from the many brain regions to which they project (Hensler, 2006). Much of the serotonergic signaling is in the form of extrasynaptic volume transmission, a slower mechanism than synaptic signaling. These features support a slower, tonic modulation of brain regions. The 5-HT1A receptor is inhibitory, so increasing serotonin by inhibiting reuptake would be expected to decrease activity (Quentin et al., 2018), consistent with the ASL CBF decrease observed. Animal (Invernizzi et al., 1992) and human studies (Selvaraj et al., 2012) indicate that acute inhibition of the serotonergic raphe nuclei by 5-HT1A receptors reduces serotonergic signaling that partially offsets the increased local serotonin concentration due to reuptake inhibition in other brain regions. Since 5-HT1A receptors are present in both excitatory and inhibitory neurons of the cortex (Aznar et al., 2003;Santana et al., 2004), increased serotonin concentration may reduce overall neural activity while preserving the excitatory/inhibitory balance. In contrast, GABA signaling originates locally from interneurons (Tremblay et al., 2016) that communicate extensively with neighboring interneurons and pyramidal neurons through synapses. This local origin and synaptic transmission may enable faster response. Indeed, inhibitory signaling from GABAergic interneurons has been implicated in the coordination of neuron firing within and across regions and plays a role in the generation of network oscillatory activity (Tremblay et al., 2016). The GABA-positive allosteric modulator alprazolam may increase the coordinating function of GABAergic interneurons, leading to increased fluctuations as measured with BOLD rsfMRI ALFF. This increase of ALFF and other changes to rsfMRI in response to alprazolam may reflect reduction of the excitatory/inhibitory ratio. The coordination of activity by increased GABA signaling need not lead to altered time-averaged metabolism or the time-averaged ASL CBF coupled to it. Indeed, parvalbumin and VIP interneurons, which represents 55% of all neocortical GABAergic interneurons (Tremblay et al., 2016), have only a minor influence on CBF regulation (Krawchuk et al., 2020).

Comparison of resting-state fluctuations measured with BOLD and ASL also suggests differences in sensitivity to pharmacological challenge, though further work is needed to determine the relative contributions of technical and physiological factors to these differences. Most notably, BOLD detected a highly significant increase in fluctuation amplitude after alprazolam administration that was not detected with ASL rsCBF fluctuations. This might reflect lower inherent sensitivity of ASL to temporal fluctuations (Q. Zou et al., 2015), though detecting elevated activity across the entire cortex, as seen here with BOLD, should not require high sensitivity. ASL could also have different sensitivity to systemic physiological fluctuations that may mask or contribute to the observed increase in ALFF (Gevers et al., 2011). However, ASL is likely less sensitive to motion-induced fluctuations because of the heavy background suppression employed. The absence of any significant difference in motion during BOLD acquisitions across drug states suggests motion-induced noise is not a likely explanation. Different neurovascular coupling to inhibitory stimulation, compared with excitatory, has recently been reported (Moon et al., 2021). As described in the introduction, such altered coupling could also change the relative response of CBF and BOLD (Buxton et al., 2014;Devi et al., 2022).

BOLD rsfMRI and ASL rsCBF fluctuations also differed in the detected connectivity changes with alprazolam. ASL showed an increase in frontal, temporal, parietal, and occipital association areas that was similar for the local LC measure and the more distant IC measure, while BOLD showed a less extensive increase of LC in association cortex and then only decreases in sensory motor cortex. While these differences are less significant than the ALFF effect with alprazolam and should not be overinterpreted, the differences are consistent with the previously reported higher baseline excitatory/inhibitory ratio in association cortex and lowest ratio in motor cortex (Fotiadis et al., 2023;Goulas et al., 2021). Higher E/I ratio cortices may be more susceptible to the reduction of E/I ratio induced by alprazolam. The differences between BOLD and ASL support a very local increase in fluctuation power within only the BOLD measure.

The increased BOLD rsfMRI ALFF after alprazolam at first may seem inconsistent with its sedative effect and its relationship with inhibitory GABA signaling, but thoughtful review of the literature supports this change. Sedation or sleepiness need not imply reduced neural activity or BOLD amplitude. Elevated resting-state BOLD amplitude with increased local correlations has been reported in nonrapid eye movement sleep cycle (Tagliazucchi et al., 2013). A number of studies (Kiemes et al., 2021) have shown an inverse relationship across subjects between BOLD response to stimulus and the regional concentration of GABA as measured with MR spectroscopy, but the physiological mechanism underlying intersubject or scan differences in GABA concentration is complex. The higher GABA concentration may reflect higher receptor activity only or might covary with reduced signaling efficiency or other factors. One pharmacological study (Walter et al., 2016) showed an inverse correlation between BOLD response to a working memory task and serum concentration of diazepam, a GABA-positive allosteric modulator, but this could reflect the effect of drowsiness on task performance. A recent study assessed rsfMRI changes in response to 5 days of alprazolam administration and found widespread cortical increase of very low-frequency rsfMRI fluctuations (Wein et al., 2024) as measured by a low-frequency version of the fractional ALFF (fALFF) measure (Q.-H. Zou et al., 2008). The low-frequency analysis was motivated by an older study showing increased low-frequency power following midazolam administration (J. Kiviniemi et al., 2005). In contrast, another older study (Flodin et al., 2012) measured fractional ALFF (fALFF) including higher frequency fluctuations in subjects administered with oxazepam, another GABA-positive allosteric modulator, and reported no change. fALFF measures relative fluctuations in the targeted frequency range relative to the total power, so results may be very dependent on the chosen frequency range. These findings suggest that elevated low-frequency power in rsfMRI may be a marker for excitatory/inhibitory balance, similar to previously proposed spectral measures from electroencephalography (Gao et al., 2017).

Though our data unambiguously support sensitivity of imaging methods to neurotransmitter modulations in the brain, the study was not designed to directly assess treatment mechanisms. The participants involved were not affected by the target clinical disorders, no measures of treatment efficacy were obtained, and the single-dose intervention is not consistent with normal therapeutic regimes, though neural responses to single doses might be predictive of therapeutic responses. The time scale for therapeutic response of depressed patients to SSRIs is somewhat controversial but is at least 1 week or longer (Taylor et al., 2006). The absence of immediate response has been attributed to slower changes in receptor sensitivities and densities (Richelson, 2001). Though therapeutic response to alprazolam is faster, initial treatment is associated with nonspecific side effects, especially drowsiness, which may improve with more extended use (Schweizer et al., 1993). Our observed changes in BOLD ALFF across the cortex may partly reflect these nonspecific brain effects. The GABA-positive allosteric modulator alprazolam has been shown to attenuate hypothalamic–pituitary–adrenal response to stress (Fries et al., 2006) due in part to direct action on the adrenal gland (Grottoli et al., 2002). These changes are unlikely to affect brain studies in participants without anxiety. Nevertheless, the ability to probe and map physiological changes in response to drug administration could be a powerful tool to study on and off target effects and mechanisms of therapeutic action.

Several previous studies have investigated the impact of citalopram or related SSRIs on the brain as assessed with functional imaging. One study with ASL in 12 subjects (Chen et al., 2011) showed reduction of CBF in several regions after citalopram treatment, qualitatively consistent with our results. Another study showed decreased ASL in cortical regions and the thalamus but with poor test–retest reliability (Klomp et al., 2012). A third study showed decreased CBF in the thalamus that correlated with SPECT imaging of serotonin transporter occupancy (Schrantee et al., 2019). One report on multiple studies with ASL to monitor neurotransmitter targeted drugs appears to show significant negative correlation between 5-HT2 (but not 5-HT1A) distribution and CBF change with administration of escitalopram (an isomer of citalopram;Burke & Kratochvil, 2002), though the analysis and acquisition methods are different and sparsely detailed (Dukart et al., 2018). A related study (Viviani et al., 2012) measured ASL changes induced by a different SSRI, paroxetine, administered for 7 days prior to imaging and found a similar spatial distribution of decrease to ours, but primarily on the left side. Though differences in dose, ASL method, number of participants, and analysis method make detailed comparison difficult, there is reasonable consistency among the studies. Less consistency of methods and results is present in the BOLD rsfMRI literature. One study measured BOLD rsfMRI degree centrality following escitalopram administration and found widespread decreases (Schaefer et al., 2014). Degree centrality appears closely related to the intrinsic connectivity measure employed in our study and we observed no significant decreases. A number of other studies using a range of different seeds and cross component analyses have been reported (Arnone et al., 2018;Drew et al., 2020;Dutta et al., 2019;Howarth et al., 2021;Stewart et al., 2014). These studies mostly report reduced connectivity, though the use of different seeds and measures makes detailed comparison impossible.

Alprazolam effects on CBF and BOLD rsfMRI have also been previously reported. In one earlier study (Roy-Byrne et al., 1993), the effect of intravenous infusion of alprazolam on CBF was assessed with [^15^O]H_2_0 PET. A marked decrease in CBF across the brain was observed after infusion, but the absence of a placebo control and the use of IV infusion make comparison difficult. Another early PET study evaluated CBF changes following IV diazepam (a related benzodiazepine) and showed modest cortical decreases in CBF for placebo but also a less than 10% decrease of CBF relative to placebo (Mathew & Wilson, 1991). It appears plausible that the detection of widespread decreases in CBF may be dependent on study design, drug dose, route of administration, and subject stress. In a previous ASL study, an increase in nucleus accumbens relative CBF and a reduced relative CBF in thalamus following alprazolam administration were reported (Wolf et al., 2013). That study included more subjects (n = 45) than the current work, including 19 unaffected relatives of schizophrenia patients, employed twice the dose, and different statistical methods. Two more studies of BOLD rsfMRI changes with alprazolam have been previously reported (Blanco-Hinojo et al., 2021;Wein et al., 2024). In one study (Blanco-Hinojo et al., 2021), increases in local correlation similar to ours were reported, but ALFF, or other measure of fluctuation amplitude, was not examined. In the second study (Wein et al., 2024), a low-frequency version of fALFF was found to be elevated across the cortex, as previously discussed.

Our design included both placebo control and a pretreatment scan. The pretreatment scan is not formally necessary but can help control for differences in physiological state across days that are unrelated to the drug treatment. The placebo control was important and particularly impactful for the alprazolam study. Widespread significant reductions in ASL CBF were observed when comparing post- with pretreatment scans. However, when controlled for the effect of placebo, these reductions decreased to below significance. The case for employing a pretreatment scan was less compelling, however. Similar results were obtained with and without the pretreatment scans included in the analysis, and significance levels were overall slightly higher when the pretreatment scan was not included in the contrast. Our results provide little support for the benefit of pretreatment scans.

The widespread cortical distribution of the BOLD rsfMRI ALFF increase with alprazolam raises some questions about the mechanism and its specificity to the brain. Though this widespread ALFF increase is consistent with the widespread distribution of GABA-A receptors, a number of potential systematic errors may also tend toward widespread effects. Systemic physiological fluctuations of cardiac and respiratory function can modulate the BOLD signal (Chang et al., 2009), voluntary and involuntary motion can add noise which may alter ALFF (Turner et al., 2013), and drugs may have widespread direct effects on vascular regulation. Each of these confounds might be expected to have a fairly uniform distribution across cortex. While these causes are possible, they seem less likely than a simple neural effect. Cardiac and respiratory rates were not significantly different between either drug and placebo. Greater participant motion after alprazolam might be expected due to the drowsiness it may induce, but no statistical difference in motion variables was observed.

Our study is not without limitations. It did not evaluate dose response and was limited to a single dose. Differences in dose across studies could partially explain discrepancies between studies. For example,Schaefer et al. (2014)administered 20 mg of escitalopram which, because of its use of only the active isomer of the drug (Burke & Kratochvil, 2002), effectively doubles the dose from our 20 mg of citalopram. Our 0.5 mg dose of alprazolam was 33% lower than in a rsfMRI study with similar findings and 50% lower than in an earlier publication with ASL (Wolf et al., 2013) that showed elevation of nucleus accumbens perfusion. Another recent rsfMRI study used a 0.5 mg dose repeated three times daily over several days prior to imaging (Wein et al., 2024).

Although prior work has suggested that in the resting-state BOLD ALFF is correlated with CBF and metabolism (Li et al., 2012;Tomasi et al., 2013), albeit with spatial variations (Mantini et al., 2007), our current findings show that BOLD ALFF–CBF relationships can also be dissociated when neurotransmitter systems are modulated pharmacologically. Prior work has also shown spatial heterogeneity in resting-state BOLD and CBF responses to pharmacological challenges (Khalili-Mahani et al., 2014), suggesting that spatial variations in both neurotransmitter receptors and cerebrovascular architecture contribute to observed phMRI responses across fMRI modalities. Together, these findings suggest the need for further characterization of fMRI responses to neuroactive drugs and a multimodal approach to phMRI.

## 5 Conclusion

Functional MRI methods have differential sensitivity to drugs targeting neurotransmitter systems. Our findings indicate strikingly different responses of BOLD rsfMRI and ASL for citalopram and alprazolam modulations. Our results contribute to evidence suggesting the excitatory/inhibitory ratio as a driver of local BOLD rsfMRI amplitude and frequency spectrum. ASL time-averaged CBF, in contrast, may reflect the combined metabolic activity of excitatory and inhibitory neurons. Thus, different fMRI methods and measures may be better suited to capture different neurotransmitter modulations, and combined BOLD and ASL studies may be advantageous for phMRI studies.

## Supplementary Material

Supplementary Material

## Data Availability

Anonymized imaging data will be shared upon request from any interested and qualified investigator after completing a Data Sharing Agreement with Beth Israel Deaconess Medical Center.
