# Supplementary material for: Pharmacological manipulation of neurotransmitter activity induces disparate effects on cerebral blood flow and resting-state fluctuations
Source: Imaging Neurosci (Camb). 2024 Nov 20;2:imag-2-00370. doi: 10.1162/imag_a_00370 (PMC12315738; doi:10.1162/imag_a_00370)
Supplement: Supplementary Material [file imag_a_00370-supp.pdf]

Supplementary material

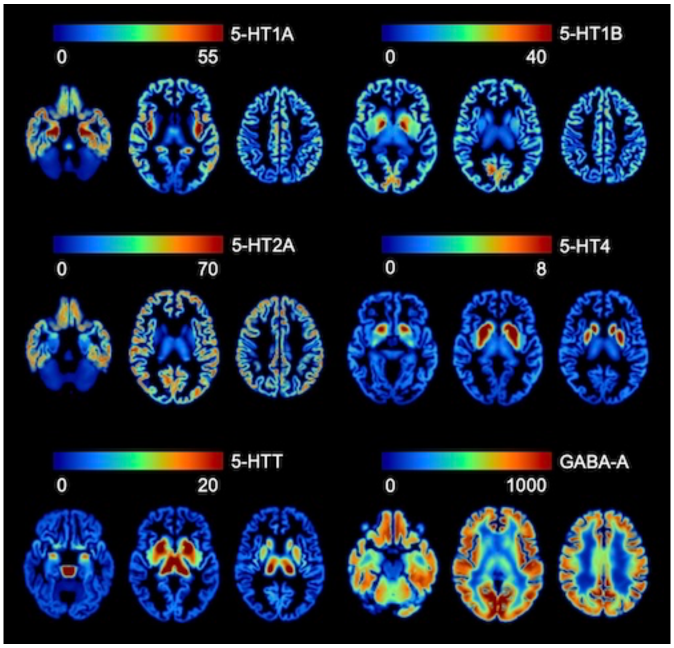

Supplementary figure 1: Templates of serotonergic and GABAergic receptors / transporters density profiles. Adapted from Beliveau et al. (2017) and Nørgaard et al. (2021)

	Conditions	z value	p-value
**Motion parameters**	Citalopram $_{post}$ > placebo $_{post}$	-0.03	0.980
	Citalopram $_{post\text{-}pre}$ > placebo $_{post\text{-}pre}$	0.59	0.555
	Alprazolam $_{post}$ > placebo $_{post}$	0.06	0.950
	Alprazolam $_{post\text{-}pre}$ > placebo $_{post\text{-}pre}$	0.22	0.830
**Cardiac rate**	Citalopram $_{post}$ > placebo $_{post}$	0.117	0.907
	Citalopram $_{post\text{-}pre}$ > placebo $_{post\text{-}pre}$	1.254	0.21
	Alprazolam $_{post}$ > placebo $_{post}$	-0.895	0.371
	Alprazolam $_{post\text{-}pre}$ > placebo $_{post\text{-}pre}$	0.879	0.379
**Respiratory rate**	Citalopram $_{post}$ > placebo $_{post}$	0.851	0.395
	Citalopram $_{post\text{-}pre}$ > placebo $_{post\text{-}pre}$	0.732	0.464
	Alprazolam $_{post}$ > placebo $_{post}$	0.872	0.383
	Alprazolam $_{post\text{-}pre}$ > placebo $_{post\text{-}pre}$	1.151	0.25

Supplementary table 1: Linear mixed-effect model assessing after drug intake (1) the relationship between BOLD rsfMRI ALFF maps and motion parameters and (2) the differences in cardiac and respiratory rates between scans

Drug	Clusters	Size (voxels)	Mass p-FDR	Brain regions
Citalopram	Positive cluster 1	2476	0.046	Cerebellum
	Negative cluster 1	1226	0.046	Left temporal pole<br>Left hippocampus<br>Left amygdala<br>Left parahippocampal gyrus, anterior division<br>Left insular cortex<br>Left putamen
	Negative cluster 2	1085	0.046	Right hippocampus<br>Right amygdala<br>Right parahippocampal gyrus, anterior division<br>Right insular cortex<br>Right temporal pole
	Negative cluster 3	1009	0.046	Right postcentral gyrus<br>Right precentral gyrus<br>Right central opercular cortex<br>Right middle temporal gyrus, posterior division<br>Right planum temporale<br>Right supramarginal gyrus, anterior division<br>Right superior temporal gyrus, posterior division<br>Right Heschl's gyrus

Supplementary table 2: Significant clusters of mean globally-normalized time-averaged CBF $_{post}$ changes in the citalopram group compared to the placebo group

Drug	Clusters	Size (voxels)	Mass p-FDR	Brain regions
Citalopram	Positive cluster 1	1910	0.018	Cerebellum
	Negative cluster 1	2109	0.018	Left temporal pole<br>Left central opercular cortex<br>Left insular cortex<br>Left hippocampus<br>Left amygdala<br>Left postcentral gyrus<br>Left frontal orbital cortex<br>Left putamen

	Negative cluster 2	1625	0.018	Right hippocampus<br>Right temporal pole<br>Right amygdala<br>Right parahippocampal gyrus, posterior division<br>Right insular cortex<br>Right inferior temporal gyrus, anterior division<br>Right frontal orbital cortex<br>Right parahippocampal gyrus,<br>anterior division

Supplementary table 3: Significant clusters of mean globally-normalized time-averaged CBF $_{post\text{-}pre}$ in the citalopram group compared to the placebo group

Supplementary table 4: Time-averaged CBF changes with citalopram intake in neurotransmitters regions

Neurotransmitter receptors / transporters	Conditions	z value	p-value
5-HTT	Citalopram $_{post}$ > placebo $_{post}$	0.88	0.381
	Citalopram $_{post\text{-}pre}$ > placebo $_{post\text{-}pre}$	0.56	0.574
5-HT1A	Citalopram $_{post}$ > placebo $_{post}$	0.62	0.535
	Citalopram $_{post\text{-}pre}$ > placebo $_{post\text{-}pre}$	0.39	0.695
5-HT1B	Citalopram $_{post}$ > placebo $_{post}$	0.84	0.401
	Citalopram $_{post\text{-}pre}$ > placebo $_{post\text{-}pre}$	0.62	0.534
5-HT2A	Citalopram $_{post}$ > placebo $_{post}$	0.81	0.42
	Citalopram $_{post\text{-}pre}$ > placebo $_{post\text{-}pre}$	0.60	0.552
5-HT4	Citalopram $_{post}$ > placebo $_{post}$	0.76	0.447
	Citalopram $_{post\text{-}pre}$ > placebo $_{post\text{-}pre}$	0.52	0.603
GABA-A	Citalopram $_{post}$ > placebo $_{post}$	1.10	0.273
	Citalopram $_{post\text{-}pre}$ > placebo $_{post\text{-}pre}$	0.76	0.447

Supplementary table 4: Linear mixed-effect model assessing the relationship between time-averaged CBF (global signal included) and neurotransmitters receptors / transporters density profiles after citalopram intake

Supplementary table 5: ASL rsCBF fluctuations ALFF changes with citalopram intake in neurotransmitters regions

Neurotransmitter receptors / transporters	Conditions	z value	p-value
5-HTT	Citalopram $_{post}$ > placebo $_{post}$	1.33	0.183
	Citalopram $_{post\text{-}pre}$ > placebo $_{post\text{-}pre}$	-0.53	0.593

5-HT1A	Citalopram $_{post}$ > placebo $_{post}$	1.37	0.170
	Citalopram $_{post-pre}$ > placebo $_{post-pre}$	-0.50	0.618
5-HT1B	Citalopram $_{post}$ > placebo $_{post}$	1.33	0.183
	Citalopram $_{post-pre}$ > placebo $_{post-pre}$	-0.59	0.554
5-HT2A	Citalopram $_{post}$ > placebo $_{post}$	1.39	0.165
	Citalopram $_{post-pre}$ > placebo $_{post-pre}$	-0.54	0.590
5-HT4	Citalopram $_{post}$ > placebo $_{post}$	1.33	0.185
	Citalopram $_{post-pre}$ > placebo $_{post-pre}$	-0.56	0.576
GABA-A	Citalopram $_{post}$ > placebo $_{post}$	1.41	0.158
	Citalopram $_{post-pre}$ > placebo $_{post-pre}$	-0.59	0.554

Supplementary table 5: Linear mixed-effect model assessing the relationship between ASL rsCBF fluctuations ALFF maps and neurotransmitters receptors / transporters density profiles after citalopram intake

Supplementary table 6: ASL rsCBF fluctuations IC changes with citalopram intake in neurotransmitters regions

**Neurotransmitter receptors / transporters**	**Conditions**	**z value**	**p-value**
5-HTT	Citalopram $_{post}$ > placebo $_{post}$	0.86	0.389
	Citalopram $_{post-pre}$ > placebo $_{post-pre}$	-0.07	0.945
5-HT1A	Citalopram $_{post}$ > placebo $_{post}$	0.91	0.364
	Citalopram $_{post-pre}$ > placebo $_{post-pre}$	-0.21	0.835
5-HT1B	Citalopram $_{post}$ > placebo $_{post}$	0.87	0.386
	Citalopram $_{post-pre}$ > placebo $_{post-pre}$	-0.19	0.848
5-HT2A	Citalopram $_{post}$ > placebo $_{post}$	0.90	0.368
	Citalopram $_{post-pre}$ > placebo $_{post-pre}$	-0.21	0.836
5-HT4	Citalopram $_{post}$ > placebo $_{post}$	0.90	0.370
	Citalopram $_{post-pre}$ > placebo $_{post-pre}$	-0.16	0.872
GABA-A	Citalopram $_{post}$ > placebo $_{post}$	0.89	0.375
	Citalopram $_{post-pre}$ > placebo $_{post-pre}$	-0.21	0.832

Supplementary table 6: Linear mixed-effect model assessing the relationship between ASL rsCBF fluctuations IC maps and neurotransmitters receptors / transporters density profiles after citalopram intake

Supplementary table 7: ASL rsCBF fluctuations LC changes with citalopram intake in neurotransmitters regions

**Neurotransmitter receptors / transporters**	**Conditions**	**z value**	**p-value**
5-HTT	Citalopram $_{post}$ > placebo $_{post}$	0.62	0.535
	Citalopram $_{post-pre}$ > placebo $_{post-pre}$	-0.30	0.765
5-HT1A	Citalopram $_{post}$ > placebo $_{post}$	0.86	0.390

	Citalopram $_{post\text{-}pre}$ > placebo $_{post\text{-}pre}$	-0.44	0.663
5-HT1B	Citalopram $_{post}$ > placebo $_{post}$	0.77	0.443
	Citalopram $_{post\text{-}pre}$ > placebo $_{post\text{-}pre}$	-0.47	0.642
5-HT2A	Citalopram $_{post}$ > placebo $_{post}$	0.85	0.398
	Citalopram $_{post\text{-}pre}$ > placebo $_{post\text{-}pre}$	-0.48	0.631
5-HT4	Citalopram $_{post}$ > placebo $_{post}$	0.72	0.467
	Citalopram $_{post\text{-}pre}$ > placebo $_{post\text{-}pre}$	-0.41	0.680
GABA-A	Citalopram $_{post}$ > placebo $_{post}$	0.77	0.440
	Citalopram $_{post\text{-}pre}$ > placebo $_{post\text{-}pre}$	-0.546	0.585

Supplementary table 7: Linear mixed-effect model assessing the relationship between ASL rsCBF fluctuations LC maps and neurotransmitters receptors / transporters density profiles after citalopram intake

Supplementary table 8: BOLD rsfMRI ALFF changes with citalopram intake in neurotransmitters regions

Neurotransmitter receptors / transporters	Conditions	z value	p-value
5-HTT	Citalopram $_{post}$ > placebo $_{post}$	0.68	0.496
	Citalopram $_{post\text{-}pre}$ > placebo $_{post\text{-}pre}$	-0.16	0.874
5-HT1A	Citalopram $_{post}$ > placebo $_{post}$	0.87	0.382
	Citalopram $_{post\text{-}pre}$ > placebo $_{post\text{-}pre}$	-0.15	0.878
5-HT1B	Citalopram $_{post}$ > placebo $_{post}$	0.82	0.415
	Citalopram $_{post\text{-}pre}$ > placebo $_{post\text{-}pre}$	-0.21	0.836
5-HT2A	Citalopram $_{post}$ > placebo $_{post}$	0.88	0.381
	Citalopram $_{post\text{-}pre}$ > placebo $_{post\text{-}pre}$	-0.20	0.842
5-HT4	Citalopram $_{post}$ > placebo $_{post}$	0.80	0.422
	Citalopram $_{post\text{-}pre}$ > placebo $_{post\text{-}pre}$	-0.18	0.859
GABA-A	Citalopram $_{post}$ > placebo $_{post}$	0.77	0.444
	Citalopram $_{post\text{-}pre}$ > placebo $_{post\text{-}pre}$	-0.14	0.891

Supplementary table 8: Linear mixed-effect model assessing the relationship between BOLD rsfMRI ALFF maps and neurotransmitters receptors / transporters density profiles after citalopram intake

Supplementary table 9: BOLD rsfMRI IC changes with citalopram intake in neurotransmitters regions

Neurotransmitter receptors / transporters	Conditions	z value	p-value
5-HTT	Citalopram $_{post}$ > placebo $_{post}$	-0.59	0.555
	Citalopram $_{post\text{-}pre}$ > placebo $_{post\text{-}pre}$	-1.35	0.177
5-HT1A	Citalopram $_{post}$ > placebo $_{post}$	-0.21	0.833
	Citalopram $_{post\text{-}pre}$ > placebo $_{post\text{-}pre}$	-1.16	0.244

5-HT1B	Citalopram $_{post}$ > placebo $_{post}$	-0.54	0.590
	Citalopram $_{post-pre}$ > placebo $_{post-pre}$	-1.26	0.208
5-HT2A	Citalopram $_{post}$ > placebo $_{post}$	-0.48	0.629
	Citalopram $_{post-pre}$ > placebo $_{post-pre}$	-1.24	0.216
5-HT4	Citalopram $_{post}$ > placebo $_{post}$	-0.27	0.789
	Citalopram $_{post-pre}$ > placebo $_{post-pre}$	-1.12	0.264
GABA-A	Citalopram $_{post}$ > placebo $_{post}$	-0.82	0.415
	Citalopram $_{post-pre}$ > placebo $_{post-pre}$	-1.30	0.193

Supplementary table 9: Linear mixed-effect model assessing the relationship between BOLD rsfMRI IC maps and neurotransmitters receptors / transporters density profiles after citalopram intake

Supplementary table 10: BOLD rsfMRI LC changes with citalopram intake in neurotransmitters regions

**Neurotransmitter receptors / transporters**	**Conditions**	**z value**	**p-value**
5-HTT	Citalopram $_{post}$ > placebo $_{post}$	0.09	0.928
	Citalopram $_{post-pre}$ > placebo $_{post-pre}$	-0.97	0.334
5-HT1A	Citalopram $_{post}$ > placebo $_{post}$	0.03	0.979
	Citalopram $_{post-pre}$ > placebo $_{post-pre}$	-0.93	0.354
5-HT1B	Citalopram $_{post}$ > placebo $_{post}$	-0.13	0.893
	Citalopram $_{post-pre}$ > placebo $_{post-pre}$	-1.06	0.289
5-HT2A	Citalopram $_{post}$ > placebo $_{post}$	-0.17	0.864
	Citalopram $_{post-pre}$ > placebo $_{post-pre}$	-1.06	0.291
5-HT4	Citalopram $_{post}$ > placebo $_{post}$	0.18	0.859
	Citalopram $_{post-pre}$ > placebo $_{post-pre}$	-0.91	0.365
GABA-A	Citalopram $_{post}$ > placebo $_{post}$	-0.45	0.650
	Citalopram $_{post-pre}$ > placebo $_{post-pre}$	-1.09	0.276

Supplementary table 10: Linear mixed-effect model assessing the relationship between BOLD rsfMRI LC maps and neurotransmitters receptors / transporters density profiles after citalopram intake

**Drug**	**Clusters**	**Size (voxels)**	**Mass p-FDR**	**Brain regions**
Alprazolam	Positive cluster 1	68187	< 0.000001	Bilateral precentral gyri<br>Precuneus cortex<br>Bilateral superior frontal gyri<br>Bilateral frontal poles<br>Cingulate gyrus<br>Bilateral postcentral gyri<br>Bilateral lateral occipital cortices, superior division<br>Bilateral middle frontal gyri

				Bilateral lingual gyri<br>Bilateral paracingulate gyri<br>Cerebellum<br>Bilateral supplementary motor areas<br>Bilateral superior parietal lobules<br>Bilateral intracalcarine cortices<br>Bilateral occipital fusiform gyri<br>Bilateral temporal occipital fusiform cortices<br>Bilateral insular cortices<br>Bilateral cuneal cortices<br>Bilateral angular gyri<br>Bilateral occipital poles<br>Bilateral lateral occipital cortex, inferior division<br>Bilateral central opercular cortex<br>Bilateral middle temporal gyri, posterior division<br>Right putamen<br>Bilateral middle temporal gyri, temporooccipital part<br>Bilateral planum temporale<br>Bilateral supramarginal gyri, posterior division<br>Bilateral superior temporal gyri, posterior division<br>Bilateral parietal operculum cortices<br>Bilateral temporal poles<br>Bilateral superior temporal gyrus, anterior division<br>Bilateral temporal fusiform cortices, posterior division<br>Bilateral planum polare<br>Bilateral Heschl’s gyrus<br>Bilateral inferior temporal gyrus, temporooccipital part<br>Bilateral supramarginal gyrus, anterior division<br>Right frontal orbital cortex

				Bilateral inferior frontal gyri, pars opercularis<br>Bilateral thalami<br>Bilateral supracalcarine cortices<br>Right hippocampus<br>Bilateral parahippocampal gyri, posterior division<br>Left middle temporal gyrus, anterior division<br>Left inferior frontal gyrus, pars triangularis<br>Right caudate<br>Right pallidum<br>Left inferior temporal gyrus, posterior division<br>Fontal medial cortex<br>Bilateral frontal operculum cortices<br>Brainstem
	Positive cluster 2	996	0.044040	Left putamen<br>Left frontal orbital cortex<br>Left pallidum<br>Left temporal pole<br>Left insular cortex<br>Left amygdala<br>Left caudate

Supplementary table 11: Significant clusters of BOLD rsfMRI ALFF changes in the afternoon in the alprazolam group compared to the placebo group

Supplementary table 12: ASL rsCBF fluctuations ALFF changes with alprazolam intake in neurotransmitters regions

**Neurotransmitter receptors / transporters**	**Conditions**	**z value**	**p-value**
5-HTT	Alprazolam $_{post}$ > placebo $_{post}$	0.13	0.900
	Alprazolam $_{post - pre}$ > placebo $_{post - pre}$	-0.38	0.704
5-HT1A	Alprazolam $_{post}$ > placebo $_{post}$	0.23	0.817
	Alprazolam $_{post - pre}$ > placebo $_{post - pre}$	-0.29	0.773
5-HT1B	Alprazolam $_{post}$ > placebo $_{post}$	0.35	0.724
	Alprazolam $_{post - pre}$ > placebo $_{post - pre}$	-0.20	0.839
5-HT2A	Alprazolam $_{post}$ > placebo $_{post}$	0.39	0.696
	Alprazolam $_{post - pre}$ > placebo $_{post - pre}$	-0.18	0.861
5-HT4	Alprazolam $_{post}$ > placebo $_{post}$	0.25	0.801

	Alprazolam $_{post - pre}$ > placebo $_{post - pre}$	-0.32	0.752
GABA-A	Alprazolam $_{post}$ > placebo $_{post}$	0.36	0.717
	Alprazolam $_{post - pre}$ > placebo $_{post - pre}$	-0.19	0.847

Supplementary table 12: Linear mixed-effect model assessing the relationship between ASL rsCBF fluctuations ALFF maps and neurotransmitters receptors / transporters density profiles after alprazolam intake

Drug	Clusters	Size (voxels)	Mass p-FDR	Brain regions
Alprazolam	Positive cluster 1	7987	0.001840	Cerebellum<br>Left temporal pole<br>Bilateral lingual gyri<br>Left middle temporal gyrus, anterior division<br>Left angular gyrus<br>Left middle temporal gyrus, posterior division<br>Left inferior temporal gyrus, anterior division<br>Left hippocampus<br>Left superior temporal gyrus, anterior division<br>Left supramarginal gyrus, posterior division<br>Precuneus cortex<br>Left temporal fusiform cortex, posterior division<br>Cingulate gyrus, posterior division<br>Left parahippocampal gyrus, posterior division<br>Left planum polare<br>Brainstem<br>Left superior temporal gyrus, posterior division<br>Left temporal fusiform cortex, anterior division<br>Left lateral occipital cortex, superior division<br>Left temporal occipital fusiform cortex<br>Left parahippocampal gyrus, anterior division

				Left middle temporal gyrus, temporooccipital part<br>Left amygdala<br>Left planum temporale
	Positive cluster 2	2946	0.008442	Right middle temporal gyrus, posterior division<br>Right hippocampus<br>Right temporal pole<br>Right superior temporal gyrus, posterior division<br>Right planum polare<br>Right inferior temporal gyrus, anterior division<br>Right middle temporal gyrus, anterior division<br>Right insular cortex<br>Right amygdala<br>Right superior temporal gyrus, anterior division<br>Right thalamus<br>Right parahippocampal gyrus, anterior division<br>Right parahippocampal gyrus, posterior division<br>Right temporal fusiform cortex, anterior division<br>Right frontal orbital cortex
	Positive cluster 3	2527	0.008442	Cingulate gyrus<br>Bilateral precentral gyri<br>Bilateral supplementary motor areas<br>Left postcentral gyrus<br>Right paracingulate gyrus
	Positive cluster 4	2010	0.008442	Bilateral frontal poles

Supplementary table 13: Significant clusters of BOLD rsfMRI LC changes in the afternoon in the alprazolam group compared to the placebo group

Supplementary table 14: BOLD rsfMRI IC changes with alprazolam intake in neurotransmitters regions

**Neurotransmitter receptors / transporters**	**Conditions**	**z value**	**p-value**
5-HTT	Alprazolam $_{post}$ > placebo $_{post}$	1.01	0.314

	Alprazolam $_{post - pre}$ > placebo $_{post - pre}$	0.67	0.502
5-HT1A	Alprazolam $_{post}$ > placebo $_{post}$	1.39	0.163
	Alprazolam $_{post - pre}$ > placebo $_{post - pre}$	0.86	0.388
5-HT1B	Alprazolam $_{post}$ > placebo $_{post}$	1.03	0.305
	Alprazolam $_{post - pre}$ > placebo $_{post - pre}$	0.73	0.466
5-HT2A	Alprazolam $_{post}$ > placebo $_{post}$	1.14	0.252
	Alprazolam $_{post - pre}$ > placebo $_{post - pre}$	0.81	0.421
5-HT4	Alprazolam $_{post}$ > placebo $_{post}$	1.32	0.187
	Alprazolam $_{post - pre}$ > placebo $_{post - pre}$	0.91	0.362
GABA-A	Alprazolam $_{post}$ > placebo $_{post}$	0.90	0.368
	Alprazolam $_{post - pre}$ > placebo $_{post - pre}$	0.75	0.454

Supplementary table 14: Linear mixed-effect model assessing the relationship between BOLD rsfMRI IC maps and neurotransmitters receptors / transporters density profiles after alprazolam intake

Drug	Clusters	Size (voxels)	Mass p-FDR	Brain regions
Alprazolam	Negative cluster 1	2057	0.026655	Right postcentral gyrus<br>Right precentral gyrus<br>Right superior parietal lobule
	Negative cluster 2	1162	0.047473	Left postcentral gyrus<br>Left precentral gyrus

Supplementary table 15: Significant clusters of BOLD rsfMRI IC changes in the afternoon in the alprazolam group compared to the placebo group

Drug	Clusters	Size (voxels)	Mass p-FDR	Brain regions
Alprazolam	Positive cluster 1	3444	0.027552	Left lateral occipital cortex, inferior division<br>Left middle temporal gyrus, temporooccipital part<br>Left occipital fusiform gyrus<br>Left lateral occipital cortex, superior division<br>Left temporal occipital fusiform cortex<br>Left inferior temporal gyrus, temporooccipital part<br>Cerebellum<br>Left inferior temporal gyrus, posterior division<br>Left middle temporal gyrus, posterior division

				Left angular gyrus<br>Left occipital pole
	Positive cluster 2	3315	0.027552	Left middle frontal gyrus<br>Bilateral superior frontal gyri<br>Bilateral frontal poles<br>Left paracingulate gyrus<br>Left inferior frontal gyrus, pars opercularis<br>Left inferior frontal gyrus, pars triangularis
	Positive cluster 3	3195	0.027552	Bilateral frontal poles<br>Bilateral paracingulate gyri<br>Bilateral superior frontal gyri
	Positive cluster 4	2438	0.031422	Right lateral occipital cortex, inferior division<br>Right lateral occipital cortex, superior division<br>Right middle temporal gyrus, temporooccipital part<br>Right occipital fusiform gyrus<br>Right inferior temporal gyrus, temporooccipital part<br>Right angular gyrus<br>Right temporal occipital fusiform cortex<br>Right occipital pole

Supplementary table 16: Significant clusters of ASL rsCBF fluctuations LC changes in the afternoon in the alprazolam group compared to the placebo group

Drug	Clusters	Size (voxels)	Mass p-FDR	Brain regions
Alprazolam	Positive cluster 1	8980	0.020531	Bilateral frontal poles<br>Bilateral superior frontal gyri<br>Left middle frontal gyrus<br>Left temporal pole<br>Left inferior frontal gyrus, pars opercularis<br>Left inferior frontal gyrus, pars triangularis<br>Left precentral gyrus<br>Bilateral paracingulate gyri<br>Left superior temporal gyrus, anterior division

				Left middle temporal gyrus, anterior division<br>Frontal medial cortex<br>Left middle temporal gyrus, posterior division<br>Left inferior temporal gyrus, anterior division<br>Left planum polare<br>Bilateral frontal orbital cortices<br>Left superior temporal gyrus, posterior division<br>Left central opercular cortex
	Positive cluster 2	3820	0.020531	Left lateral occipital cortex, inferior division<br>Left lateral occipital cortex, superior division<br>Left middle temporal gyrus, temporooccipital part<br>Cerebellum<br>Left superior parietal lobule<br>Left angular gyrus<br>Left occipital fusiform gyrus<br>Left inferior temporal gyrus, temporooccipital part<br>Left supramarginal gyrus, posterior division<br>Left temporal occipital fusiform cortex<br>Precuneus cortex<br>Left occipital pole<br>Left postcentral gyrus
	Positive cluster 3	2850	0.020531	Right lateral occipital cortex, inferior division<br>Right lateral occipital cortex, superior division<br>Right inferior temporal gyrus, temporooccipital part<br>Right occipital pole<br>Right occipital fusiform gyrus<br>Right middle temporal gyrus, temporooccipital part<br>Right middle temporal gyrus, posterior division

				Right temporal occipital fusiform cortex<br>Cerebellum<br>Right temporal fusiform cortex, posterior division

Supplementary table 17: Significant clusters of ASL rsCBF fluctuations IC changes in the afternoon in the alprazolam group compared to the placebo group

Supplementary table 18: ASL rsCBF fluctuations LC changes with alprazolam intake in neurotransmitters regions

Neurotransmitter receptors / transporters	Conditions	z value	p-value
5-HTT	Alprazolam $_{post}$ > placebo $_{post}$	1.32	0.186
	Alprazolam $_{post - pre}$ > placebo $_{post - pre}$	0.51	0.608
5-HT1A	Alprazolam $_{post}$ > placebo $_{post}$	1.74	*0.082*
	Alprazolam $_{post - pre}$ > placebo $_{post - pre}$	0.81	0.419
5-HT1B	Alprazolam $_{post}$ > placebo $_{post}$	1.83	*0.068*
	Alprazolam $_{post - pre}$ > placebo $_{post - pre}$	0.90	0.367
5-HT2A	Alprazolam $_{post}$ > placebo $_{post}$	1.95	*0.051*
	Alprazolam $_{post - pre}$ > placebo $_{post - pre}$	0.97	0.333
5-HT4	Alprazolam $_{post}$ > placebo $_{post}$	1.55	0.122
	Alprazolam $_{post - pre}$ > placebo $_{post - pre}$	0.66	0.512
GABA-A	Alprazolam $_{post}$ > placebo $_{post}$	1.92	*0.055*
	Alprazolam $_{post - pre}$ > placebo $_{post - pre}$	0.873	0.383

Supplementary table 18: Linear mixed-effect model assessing the relationship between ASL rsCBF fluctuations LC maps and neurotransmitters receptors / transporters density profiles after alprazolam intake

Supplementary table 19: ASL rsCBF fluctuations IC changes with alprazolam intake in neurotransmitters regions

Neurotransmitter receptors / transporters	Conditions	z value	p-value
5-HTT	Alprazolam $_{post}$ > placebo $_{post}$	1.62	0.105
	Alprazolam $_{post - pre}$ > placebo $_{post - pre}$	0.80	0.427
5-HT1A	Alprazolam $_{post}$ > placebo $_{post}$	1.94	0.052
	Alprazolam $_{post - pre}$ > placebo $_{post - pre}$	1.05	0.296
5-HT1B	Alprazolam $_{post}$ > placebo $_{post}$	1.98	0.047
	Alprazolam $_{post - pre}$ > placebo $_{post - pre}$	1.10	0.272
5-HT2A	Alprazolam $_{post}$ > placebo $_{post}$	2.07	0.038
	Alprazolam $_{post - pre}$ > placebo $_{post - pre}$	1.17	0.243

5-HT4	Alprazolam $_{post}$ > placebo $_{post}$	1.83	0.067
	Alprazolam $_{post - pre}$ > placebo $_{post - pre}$	0.94	0.347
GABA-A	Alprazolam $_{post}$ > placebo $_{post}$	2.01	0.045
	Alprazolam $_{post - pre}$ > placebo $_{post - pre}$	1.07	0.283

<u>Supplementary table 19</u>: Linear mixed-effect model assessing the relationship between ASL rsCBF fluctuations IC maps and neurotransmitters receptors / transporters density profiles after alprazolam intake

<u>Supplementary table 20</u>: ASL time-averaged CBF changes with alprazolam intake in neurotransmitters regions

Neurotransmitter receptors / transporters	Conditions	z value	p-value
5-HTT	Alprazolam $_{post}$ > placebo $_{post}$	-1.21	0.226
	Alprazolam $_{post - pre}$ > placebo $_{post - pre}$	-0.99	0.321
5-HT1A	Alprazolam $_{post}$ > placebo $_{post}$	-1.53	0.127
	Alprazolam $_{post - pre}$ > placebo $_{post - pre}$	-1.27	0.203
5-HT1B	Alprazolam $_{post}$ > placebo $_{post}$	-1.48	0.138
	Alprazolam $_{post - pre}$ > placebo $_{post - pre}$	-1.21	0.227
5-HT2A	Alprazolam $_{post}$ > placebo $_{post}$	-1.52	0.128
	Alprazolam $_{post - pre}$ > placebo $_{post - pre}$	-1.25	0.212
5-HT4	Alprazolam $_{post}$ > placebo $_{post}$	-1.41	0.158
	Alprazolam $_{post - pre}$ > placebo $_{post - pre}$	-1.20	0.232
GABA-A	Alprazolam $_{post}$ > placebo $_{post}$	-1.35	0.178
	Alprazolam $_{post - pre}$ > placebo $_{post - pre}$	-1.010	0.314

<u>Supplementary table 20</u>: Linear mixed-effect model assessing the relationship between time-averaged CBF (with global signal included) and neurotransmitters receptors / transporters density profiles after alprazolam intake
